# Stress and mindfulness in Parkinson’s disease – a survey in 5000 patients

**DOI:** 10.1038/s41531-020-00152-9

**Published:** 2021-01-18

**Authors:** Anouk van der Heide, Anne E. M. Speckens, Marjan J. Meinders, Liana S. Rosenthal, Bastiaan R. Bloem, Rick C. Helmich

**Affiliations:** 1 Radboud University Medical Center, Department of Neurology, Center of Expertise for Parkinson & Movement Disorders, Nijmegen, the Netherlands; 2 Donders Institute for Brain, Cognition, and Behavior, Center for Cognitive Neuroimaging, Radboud University Nijmegen, Nijmegen, the Netherlands; 3 Radboud University Medical Center, Department of Psychiatry, Center for Mindfulness, Nijmegen, the Netherlands; 4 Radboud University Medical Center, Radboud Institute for Health Science, Scientific Center for Quality of Healthcare, Nijmegen, the Netherlands; 5 Johns Hopkins School of Medicine, Department of Neurology, Baltimore, MD United States

**Keywords:** Neurological manifestations, Quality of life, Epidemiology, Parkinson's disease

## Abstract

Many Parkinson’s disease (PD) patients notice that motor symptoms worsen during stress, and experience stress-related neuropsychiatric symptoms such as anxiety and depression. Here we investigated which personal and disease characteristics are associated with perceived stress in PD, which PD symptoms are sensitive to stress, and we assessed self-reported benefits of stress-reducing strategies such as mindfulness. We sent an online survey to the Fox Insight cohort (*n* = 28,385 PD patients, *n* = 11,413 healthy controls). The survey included specific questions about the influence of stress on PD symptoms, use of stress-reducing strategies, and several validated scales measuring perceived stress, anxiety, dispositional mindfulness, rumination, and self-compassion. We received completed surveys from 5000 PD patients and 1292 controls. Patients perceived more stress than controls. Among patients, stress was correlated with increased rumination (*R* = 0.65), lower quality of life (*R* = −0.56), lower self-compassion (*R* = −0.65), and lower dispositional mindfulness (*R* = −0.48). Furthermore, patients indicated that stress significantly worsened both motor symptoms – especially tremor – and non-motor symptoms. Physical exercise was most frequently used to reduce stress (83.1%). Mindfulness was practiced by 38.7% of PD respondents, who noticed improvement in both motor and non-motor symptoms. Among non-users, 43.4% were interested in gaining mindfulness skills. We conclude that PD patients experience greater levels of stress than controls, and that stress worsens both motor and non-motor symptoms. Mindfulness may improve PD symptom severity, with the strongest effects on anxiety and depressed mood. These findings justify further controlled studies to establish the merits of mindfulness and other stress-alleviating interventions.

## Introduction

Parkinson’s disease (PD) is a neurodegenerative disorder characterized by degeneration of dopaminergic neurons in the nigrostriatal pathway, resulting in striatal dopamine depletion. The cardinal motor symptoms are bradykinesia, tremor, and rigidity, but many patients also have non-motor symptoms such as anxiety, depression, apathy, sleeping problems, or cognitive impairment^1,2^.

Both acute and chronic stress play an important role in PD^3^. Acute stress involves a physiological reaction in response to a perceived threat, in order to restore homeostatic balance^4^. In PD, acute stress may worsen motor symptoms such as freezing of gait^5^, dyskinesias^6^, and tremor^7^, and it also reduces the effect of dopaminergic medication^8^. During chronic stress, constant activation of the stress system disrupts homeostasis, thereby increasing the risk of depressive and anxiety disorders^9^. In PD, the prevalence of depression and anxiety is substantially increased^10,11^, and levels of cortisol, a marker of stress, are elevated^12^, which supports the hypothesis that PD patients are especially vulnerable to effects of stress. Recently, the covid-19 pandemic served as a major stressor worldwide, and we showed in 358 PD patients that those with higher covid-19-related stressor load experienced more PD motor and non-motor symptom severity which were likely secondary to greater psychological distress as well as lifestyle changes, in particular a lack of physical activity because patients are forced to stay more inside their house to mitigate the risk of becoming infected^13^. Chronic stress might even accelerate PD disease progression, supported by PD models in rodents showing accelerated dopaminergic neuronal loss^14^ and increased cerebral alphasynucleopathy after chronic stress^15^. It remains unclear which factors are associated with experienced stress, which PD symptoms are especially sensitive to stress, what patients do themselves to reduce stress, and what effect these strategies have.

Here, we addressed this by performing an elaborate online exploratory survey about stress in a large PD population. Our assumptions were informed by previous work, which showed that healthy people with low trait mindfulness have increased cortisol levels and are more vulnerable to social stress^16,17^. Furthermore, low self-compassion^18^ and increased rumination^19^ have been associated with increased stress. Thus, we predicted that these factors also play a role in PD. We also explored the patients’ own experience with stress-reducing interventions. We focussed specifically on mindfulness, which is defined as moment-to-moment non-judgemental awareness^20^. Mindfulness-based interventions have been shown to successfully reduce psychological distress and improve symptoms in several chronic diseases, including mental disorders^21^, cancer^22^, chronic pain^23^, and vascular disease^24^. MBI are usually offered face-to-face in groups of patients, but internet-based MBI were shown to be similarly effective in reducing psychological distress^25^. Especially in times of covid-19, such effective online solutions are essential. In PD, several studies show positive effects on clinical symptoms, albeit with somewhat inconsistent results^26^. A recent randomized controlled trial showed that eight weeks of mindfulness yoga improved motor symptoms as well as depressive and anxiety symptoms^27^. No studies explored whether the effect of mindfulness differed between symptoms. Thus, we evaluated how many patients use mindfulness in their daily lives, and also the (self-reported) effect of mindfulness on PD symptoms.

## Results

### Demographics

With a response rate of 21.4%, we received complete surveys from 5000 PD patients (48.6% women). The mean age was 67.3 years and average disease duration was 5.9 years. Importantly, 4893 of the PD respondents confirmed to have received a diagnosis from either their home doctor or their neurologist (98%). 3214 of these 5000 PD patients (64%) agreed to fill out the additional optional questions, of which 2838 completed all additional questions. In addition, 1292 controls completed the survey (mean age 60.8 years, 78.0% women). 48.4% (*n* = 625) of these controls had a 1^st^ and/or 2^nd^ degree relative, 20.9% (*n* = 270) had a spouse/partner with PD, 15.0% (*n* = 194) had a friend with PD, 6.9% (*n* = 89) was caregiver for someone with PD, 5.0% (*n* = 65) had a job related to PD, and 6.0% (*n* = 78) had no connection with PD. People who quit the survey before finishing it, were included as responders in analysis for all completed questions. Non-responders included only people who did not fill in any of the survey questions. Of all respondents, 93.9% were Caucasian, 0.5% African American, 1.0% Alaska native, 1.5% Asian, and for 3.0% race was unknown. The majority (82.6%) lived in the United States. Characteristics of the study population are shown in Table 3. PD responders scored higher on self-reported symptom severity (MDS-UPDRS-II), impairment of daily activities (PDAQ), and depressed mood (GDS) than PD non-responders. For controls, responders were significantly older and had more depressive symptoms (GDS) compared to non-responders (Table 3, columns 5 and 9). Average time between routine survey responses and the stress survey was 2.5 months (SD = 1.7) for the PDQ, 2.3 months (SD = 1.3) for the PDAQ, 3.7 months (SD = 2.8) for the GDS, 2.5 months (SD = 1.6) for the NMSQ, and 2.2 months (SD = 2.3) for the MDS-UPDRS-II.

### Comparison between PD patients and controls

Patients scored higher than controls on anxiety (PAS) (F(1,4189) = 44.2, *p* = 0.000, Cohen’s *d* = 0.23) and depressed mood (GDS) (F(1,5604) = 151.7, *p* < 0.001, Cohen’s *d* = 0.45), but lower on dispositional mindfulness (FFMQ) (F(1,4189) = 21.7, *p* = 0.000, Cohen’s *d* = 0.15), all independent of sex or age. Perceived stress (PSS) was higher in patients than in controls, but this effect was much larger for men than for women (group*sex interaction: F(1,4399)=5.7, *p* = 0.017). Self-compassion (SCS) did not differ between patients and controls (*p* = 0.150). For rumination (RRS), the main effect was not significant (*p* = 0.219), but we found a group*sex cross-over interaction (F(1,4173) = 7.6, *p* = 0.006): for women, controls scored higher than patients (*p* = 0.013), whereas for men, patients scored higher than controls (albeit non-significantly; *p* = 0.093). In general, the control responders consisted of more women and mean age was significantly lower than in the PD responders.

### Perceived effects of stress in PD patients

**Fig. 1:**
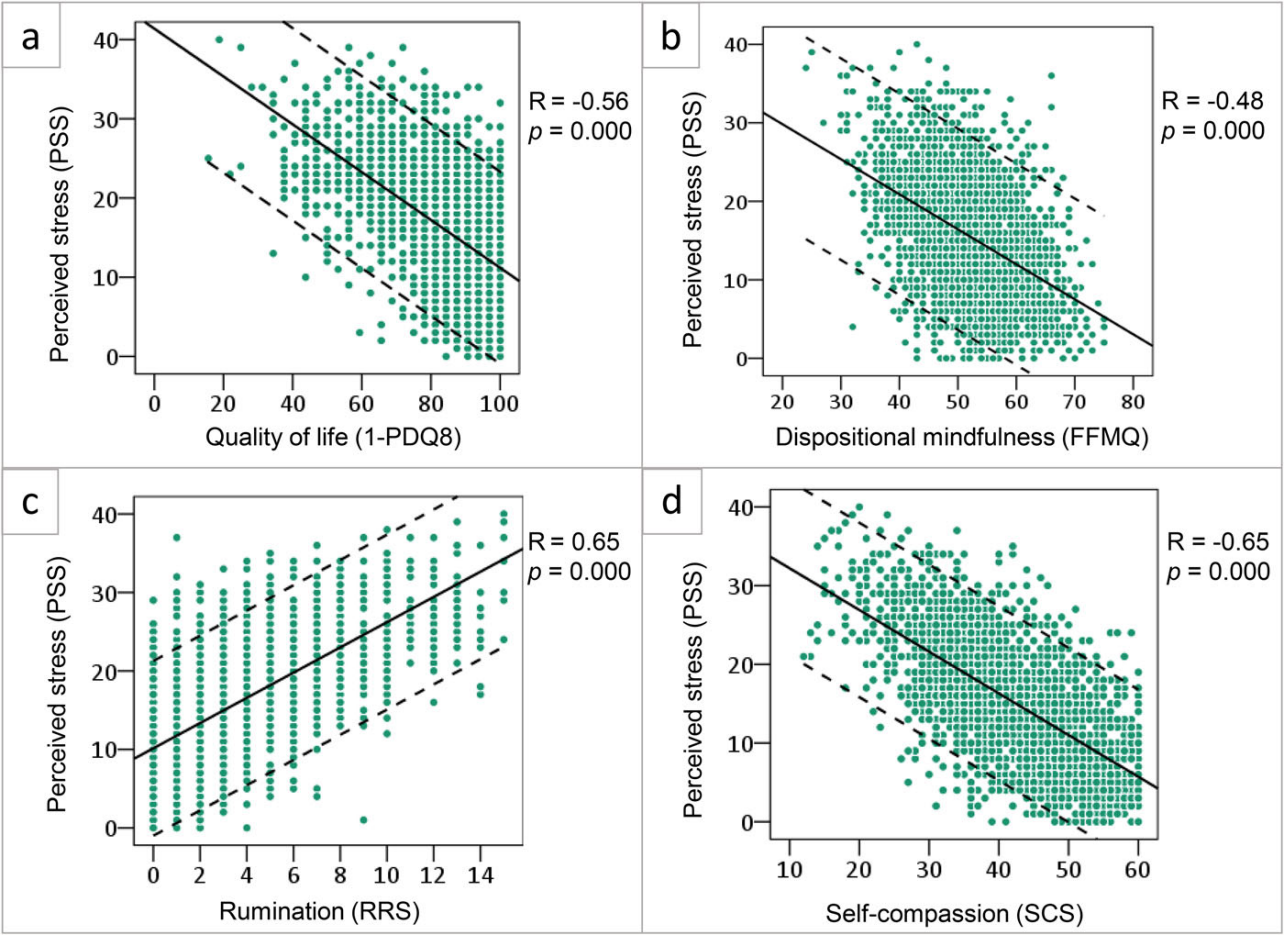
Determinants of perceived stress in Parkinson’s disease. This figure shows the relation between perceived stress as measured by the Perceived stress Scale (PSS, on the *y*-axis) and **a** quality of life measured with the Parkinson’s Disease Questionnaire (PDQ-8), *n* = 3009; **b** dispositional mindfulness measured with the Five Facet Mindfulness Questionnaire (FFMQ), *n* = 2899; **c** rumination measured with the Ruminative Response Scale (RRS), *n* = 2899; and **d** self-compassion measured with the Self Compassion Scale (SCS), *n* = 2899 (all on the *x*-axis). For the PDQ-8, we have reversed the scores (1- score), such that for each of the variables shown a higher score means a higher subjective rating. For every plot, the Pearson correlation is shown with the associated 2-tailed significance.

Scatterplots in Fig. 1a–d show that patients who perceived high levels of stress (PSS), typically ruminated more (RRS; *R* = 0.65, [95% CI 0.61–0.67]), perceived lower quality of life (PDQ-8; *R* = 0.56, [95% CI 0.52–0.58]), had low levels of self-compassion (SCS; (*R* = −0.65, [95% CI −0.67, −0.62]) and scored lower on dispositional mindfulness (FFMQ; *R* = −0.48, [95% CI −0.50, −0.43]). Furthermore, these highly stressed patients perceived higher daily life disease severity (MDS-UPDRS-II; *R* = 0.37, [95% CI 0.33–0.39]). The association between perceived stress and disease duration in the PD group was very small (*R* = 0.06, [95% CI 0.02–0.09]). All correlations were significant (*p* < 0.0083 corrected).

**Fig. 2:**
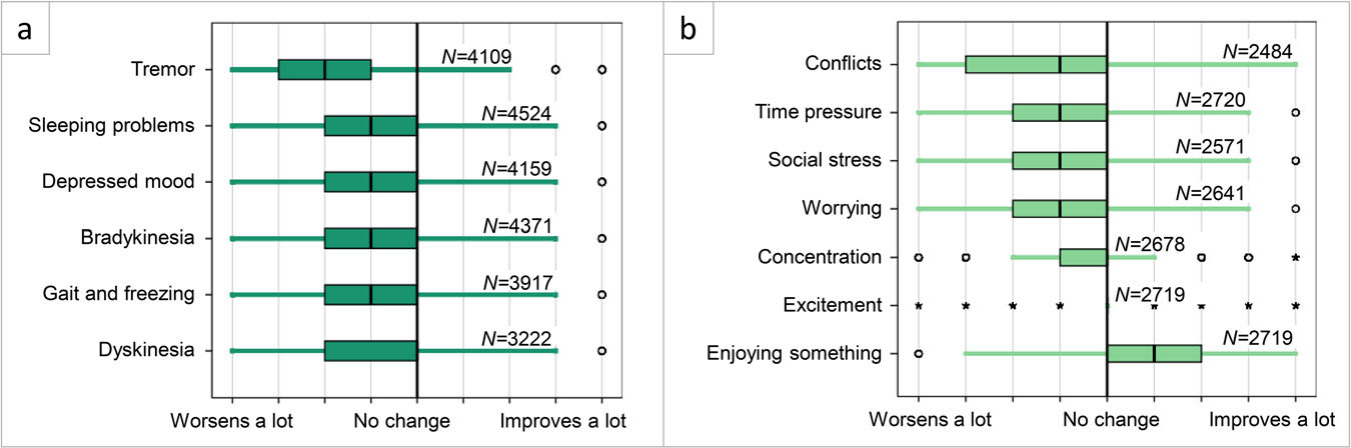
The effect of stress on Parkinson’s symptoms. The change that patients perceive on Parkinson’s symptoms during stress (Panel **a**, filled in by all PD responders (*n* = 5000)) or due to different types of stressful activities or situations (Panel **b**, part of the optional survey (*n* = 3214)). Both **a** and **b** responses were given on a 9-point scale in which 1 stood for severe worsening of symptoms, 5 stood for no change, and 9 for a lot of improvement (represented by the vertical lines). Responses are visualized in a boxplot, in which the box corresponds to 75% of responses and the tails are the remaining 25%, except outliers. Open circles show small outliers and stars show extreme outliers. Vertical lines are answer options on the 9-point scale. All shown effects are significantly different from “no change”, (*p* < 0.0083 corrected for Fig. 2a; *p* < 0.0071 corrected for Fig. 2b).

Stress worsened all PD symptoms (Fig. 2a), meaning that scores were significantly different from a score of 5 (which equals no change): tremor (mean difference from 5 (MD) = −1.93, [95% CI −1.97, −1.88]), sleeping problems (MD = −1.47, [95% CI −1.52, −1.43]), depressed mood (MD = −1.14, [95% CI −1.18, −1.09]), slowness of movement (MD = −1.13, [95% CI −1.1.17, −1.09]), gait (MD = −0.99, [95% CI −1.03, −0.94]), and dyskinesias (MD = −0.92, [95% CI −0.97, −0.87]. All changes were significant (*p* < 0.0083 corrected). In this case, an MD of −2.0 would indicate a mean score of 3.0 on the 9-point scale used for this question, where 1 stood for ‘symptom worsens a lot’ and 9 stood for ‘symptom improves a lot’. Tremor was affected most by stress: 81.8% of patients noticed worsening of their tremor during stress. When we directly compared this effect to other motor symptoms, the effect of stress on tremor was stronger than on slowness of movement, dyskinesia, or gait (*p* < 0.017 corrected). In free text fields, patients could mention other symptoms that were affected by stress. Commonly mentioned were cognitive impairment (memory problems, loss of focus, confusion, impaired executive functioning) (*n* = 188), speech and communication issues (*n* = 163), emotional symptoms (anger and frustration, anxiety, nervousness, and apathy) (*n* = 223), and pain (*n* = 63).

Patients indicated that certain activities (all involving arousal) had a particularly strong effect on overall motor symptom severity (including tremor, muscle stiffness, gait problems, or movement slowness; Fig. 2b). As above, we compared self-reported scores against a score of 5 indicating “no change”. Motor symptom severity increased due to conflicts (MD = −1.58, [95% CI −1.64, −1.53]), time pressure (MD = −1.46, [95% CI −1.52, −1.41]), social stress (MD = −1.42, [95% CI −1.47, −1.36]), worrying (MD = −1.16, [95% CI −1.21, −1.10]) and concentration (MD = −0.31, [95% CI −0.37, −0.24]). Motor symptom severity reduced when enjoying something (MD = 1.11, [95% CI 1.05–1.17]) and during excitement (e.g., after receiving good news) (MD = 0.10, [95% CI 0.04–0.15]). All changes were significant (*p* < 0.0071 corrected).

### Stress-reducing strategies

**Fig. 3:**
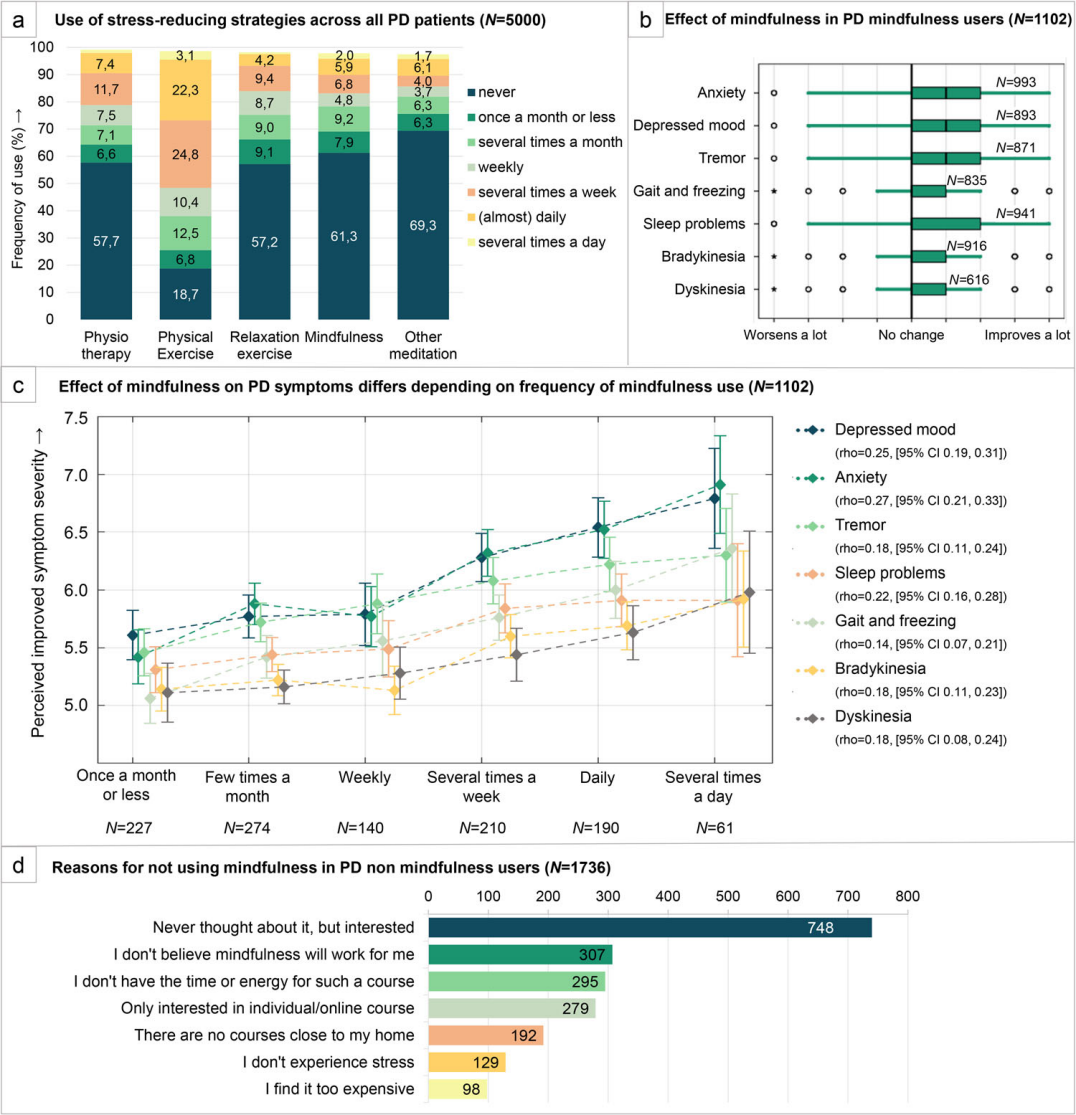
Perceived effect of mindfulness to reduce stress in PD patients. **a** The frequency with which patients use prespecified ways to reduce stress level in percentage of responders. Physical exercise could be for example walking, cycling, swimming, or sports; relaxation exercise entailed for example yoga, Pilates, or Tai Chi. **b**, **c** The change in PD symptoms that patients since they started practicing mindfulness (part of optional questions). Responses were given on a 9-point scale in which 1 stood for a lot more severity in this symptom, 5 stood for no change, and 9 for a lot of improvement. **b** Responses are visualized here in a boxplot, in which the box corresponds to 75% of responses and the tails are the remaining 25%, except outliers. Open circles show small outliers and stars show extreme outliers. Vertical lines are answer options on the 9-point scale. All shown effects are significantly different from “no change”, *p* < 0.0071 corrected. **c** The positive relationship between the frequency with which mindfulness was practiced, and the level of improvements of PD symptom severity that was experienced. Lines represent the mean improvement on every PD symptom per mindfulness practice frequency, including 95% CI of all responses. Spearman’s correlations (rho) with corresponding 95% CI for all symptoms are shown in the legend. **d** Reasons to not practice mindfulness. Patients were allowed multiple answer options (part of optional questions).

When asked how frequently they used five potential ways to lower stress, physical exercise was most often mentioned (83.1% of patients) (Fig. 3a). In addition, 38.7% of PD patients practiced mindfulness, of which 85.9% recommended this to other PD patients. Within the control group, even more people (56.4%) practiced mindfulness (*χ*^2^ = 131.8, *p* < 0.001). Interestingly, mindfulness users differed significantly from non-mindfulness users, in both PD patients and controls. On average, mindfulness users scored higher on dispositional mindfulness (FFMQ) (MD = 2.01, [95% CI 1.52–2.51]), but also higher on perceived stress (PSS) (MD = 1.28, [95% CI 0.85–1.71]) anxiety (PAS)) (MD = 1.56, [95% CI 1.03–2.10]) and rumination (RRS) (MD = 0.43, [95% CI 0.25–0.62]). All group differences were significant (*p* < 0.0071 corrected). There was no difference in self-compassion (SCS) (MD = 0.13, [95% CI −0.44, 0.69]). Within PD mindfulness users, there was no difference in disease duration (MD = −0.17, [95% CI −0.47, 0.13]) or PD medication use (*χ*^2^ = 0.9, *p* = 0.65).

Patients perceived a positive effect of mindfulness on their symptoms. Those who practiced mindfulness experienced improvement due to mindfulness for all reported symptoms (Fig. 3b), when comparing the effect against a score of 5 (no change): tremor (MD = 0.89, [95% CI 0.79–0.98]), gait (MD = 0.61, [95% CI 0.52–0.70]), slowness of movement (MD = 0.39, [95% CI 0.31–0.47]), dyskinesia (MD = 0.35, [95% CI 0.25–0.45]), depressed mood (MD = 1.03, [95% CI 0.93–1.13]), anxiety (MD = 1.04, [95% CI 0.94–1.14]), and sleeping problems (MD = 0.60, [95% CI 0.50–0.70]). All changes were significant (*p* < 0.0071 corrected). Again, an MD of −2.0 would indicate a mean score of 3.0 on the 9-point scale used for this question, where 1 stood for ‘symptom worsens a lot’ and 9 stood for ‘symptom improves a lot’. Highest effects were seen for depression and anxiety, for which, respectively, 60.2% and 64.7% noticed improvement (scores higher than 5). Interestingly, when dividing mindfulness users according to how often they reported to practice mindfulness, we showed that there is a positive correlation between the frequency of mindfulness use and the perceived improvement on Parkinson’s symptoms (Fig. 3c). People who did not practice mindfulness had various reasons for this (Fig. 3d). The most frequent response, chosen by 748 of 1736 PD responders (43.4%), was that they never thought about the option of doing mindfulness, but would be interested.

If applicable, patients could complete other strategies they had to reduce stress in free text fields. Frequently mentioned were religion (reading the bible or praying) (*n* = 265), music (listening to music, singing, or playing musical instruments) (*n* = 150), reading (*n* = 82), art/creativity (*n* = 80), anxiolytic/antidepressant medication (*n* = 75), and looking for social support (meeting or talking with friends/family) (*n* = 74).

## Discussion

In this exploratory study, we investigated whether PD patients experience more stress than controls, which personality and disease characteristics are associated with stress, which PD symptoms are especially sensitive to stress, and what patients do themselves to reduce stress. There are three main findings. First, patients scored higher than controls on anxiety (PAS), perceived stress (PSS), and depressed mood (GDS), and lower on dispositional mindfulness (FFMQ). Furthermore, patients with higher stress levels ruminated more (RRS) and scored lower on quality of life (PDQ), dispositional mindfulness (FFMQ), and self-compassion (SCS). This is in line with the hypothesis that rumination is a harmful cognitive process that generates recurrent and intrusive thoughts, which can induce and worsen perceived stress^28^. On the other hand, high self-compassion suggests people to take a balanced perspective in the face of stress and approach the situation with mindfulness^17^, thereby coping more successfully. We found a significant group*sex interaction for rumination: male patients ruminated more than male controls, whereas female patients ruminated less than female controls. We checked whether female controls were more often a spouse of PD patients and therefore more prone to rumination due to their informal care role, but this was not the case: 19.1% of male controls were spouse of someone with PD against 21.5% of female controls. Previous studies showed that, generally, women are more likely to ruminate than men^29^. However, it has not been reported before that in certain chronic diseases this might be reversed. Our control group consisted mainly of women (78%), and therefore it remains to be seen whether future studies with more equal sex-distributions replicate this finding.

Second, all PD symptoms worsened during stress, with the strongest effect on tremor. This finding fits with previous studies showing that tremor worsens during stressful conditions and during cognitive load^7,30,31^, likely due to noradrenergic effects onto the thalamus^32^. It should be noted, however, that PD patients may perceive externally observable symptoms such as tremor more easily than slowness of movement or muscle stiffness^8^, which could (partly) explain the difference between tremor and other motor symptoms. Besides tremor, respondents indicated that several other motor symptoms (freezing, gait, dyskinesias, motor slowing) also worsened under stress. Freezing can be triggered by different situations^33^, for example under time pressure or when an obstacle is encountered. In a previous study, patients reported that stress increased the difficulty to overcome freezing^5^. For movement slowness, 62.5% of PD patients in our sample noticed more slowness of movement during stressful situations, consistent with a previous study showing exaggerated deficits in fine motor control after exposure to stress-evoking stimuli^31^. However, our finding contradicts with a study that showed speeding of movement execution in PD patients who were motivated to avoid mild electric shocks^34^. This might suggest that while high levels of stress worsen motor performance, people might sometimes benefit from mild stress, and subsequently experience an improved motor performance. This is actually a well-recognized phenomenon in clinical practice, where it is commonly seen that mild stress associated with a hospital visit can lead to sometimes remarkable improvement in clinical performing, such as suppression of freezing of gait, even when this symptom is debilitating when the patient is at home^35^. As for rigidity, two relatively old studies investigated the effect of emotional and mental stress, and both found that this exaggerated rigidity in PD patients^36,37^. We did not ask respondents about rigidity in our sample since changes are very difficult to subjectively recognize by patients themselves.

Our third main finding is that many patients used several ways to reduce stress, among which physical exercise was most commonly employed (83.1%), but also mindfulness (38.7%), and that these patients experienced beneficial effects on all of their symptoms. It should be noted that this included all patients who reported that they had practiced mindfulness in the past three months, independent of how often they formally applied mindfulness exercises to reduce their stress levels. In fact, many mindfulness users reported a relatively low frequency of mindfulness exercises: out of all patients reporting to have used mindfulness in the past three months, only 53.2% practiced mindfulness on a weekly basis or more, 25.3% several times a month, and 21.5% once a month or less. The fact that mindfulness users nevertheless reported significant improvements in their symptoms may suggest that they incorporate mindfulness into their lives more informally, by paying attention to daily activities and implementing lifestyle changes that we did not quantify. For all symptoms, we found a significant positive correlation between the frequency with which mindfulness was practiced and level of experienced improvement on PD symptoms. This dose–response relationship further supports the idea that mindfulness is effective in reducing PD symptoms, although this study cannot distinguish cause from consequence: people for whom mindfulness is most effective might consequently practice it more. The beneficial effects of physical exercise fit with recent work showing that aerobic exercise (three times per week cycling during six months) attenuated motor symptom severity as compared to an active control intervention (stretching)^38^. It is unclear whether aerobic exercise improves symptom severity in PD directly, by reducing stress, or both. In healthy subjects, aerobic exercise increases the resilience to psychological stressors^39^. This suggests that stress reduction and physical exercise may be two intertwined ways in which PD patients can self-manage their symptoms. The beneficial effects of mindfulness were most pronounced on anxiety and depressive symptoms. This is in line with previous studies that tested the effect of mindfulness-based interventions in PD patients: improvements in depressed mood were seen in six out of eight studies^27,40–44^, improvement in anxiety in four out of seven studies^27,40–42,44^, and improvement of motor symptoms in two out of three studies^27,45^ that measured these symptoms. The focus of mindfulness on the present moment might discourage past-oriented ruminative thoughts, explaining the beneficial effects on mood and anxiety. Empowering PD patients to better manage their stress may be important for self-management and acceptance of the consequences of the disease. Seemingly in contrast with this, mindfulness users scored higher on dispositional mindfulness (FFMQ), but also perceived more stress. This was also shown in the US National Health Interview Survey, where people practicing mindfulness meditation reported feeling more nervous and stressed than non-users^46^, suggesting that stressful people are reaching out for non-pharmacological ways to improve their stress-related problems. Another possibility is that mindfulness users are more aware of stress, anxiety, and ruminative thoughts, and therefore score higher on self-rating scales. It is, however, not possible to disentangle cause from consequence with our survey, and further randomized controlled intervention studies are therefore necessary. Other interventions with a meditative component, like yoga and Tai Chi, are also promising candidates to be further explored with RCTs. Our findings suggest that psychological distress (anxiety + depression) could be a promising primary outcome measure for an intervention study (given the self-reported effects observed here; Fig. 3b), and that the presence of (mild-to-moderate) symptoms of psychological distress could be a useful inclusion criterion (given differences between users and non-users observed here). Remarkably, many respondents (*n* = 265) mentioned turning to religion as a stress reducing strategy. Because mindfulness-based interventions are historically derived from Buddhism, differences in religiosity might play an important role in how people perceive mindfulness-based interventions^47^. In addition, religious practice might also contribute to the state of mindfulness.

Strengths of this study are the very large sample size of 5000 patients and 1292 controls, the comprehensive nature of the questionnaire, and the novelty of the topic. Furthermore, the magnitude of the observed effects was generally large. The most important limitation is that all results are based on self-report rather than empirical measurements. As outlined above, hyperkinetic movements such as tremor may more readily access awareness than hypokinetic symptoms. In addition, minority ethnic groups were largely underrepresented in our sample. Despite digital campaigns of FoxInsight to target women and people that are older, non-white, less educated, and in worse physical health, members of the FoxInsight cohort are mostly white and live in the United States^48^. Socioeconomic and cultural variables will likely influence features that might carry stigma, such as stress, and therefore the present results cannot be generalized to all PD patients. Innovative recruitment strategies are needed to reach underrepresented groups of PD patients in future studies, and to establish diverse PD cohorts that are representative of the population as a whole. Another limitation is that the control group of the FoxInsight cohort largely consisted of people with a connection to PD, either through work or by knowing someone living with PD. This may lead to more symptoms of stress and depression in the control group, given that these symptoms are more prevalent in spouses or family members caring for PD patients than in the general population^49^. If this is the case, then the group differences we observed are actually an underestimation of real differences between PD patients and the general population. Furthermore, the PD and control group were not matched for age and sex, and they were of unequal size. The percentage of women in the control group was particularly higher than expected (78%), possibly introducing a sex bias. The lower mean age in the control group could also have led to an overestimation of group differences. However, we took this into account and all reported group differences were independent of age. As in all surveys, there is the risk of a selection bias, given the relatively low response rate of 16%. For example, it is remarkable that almost 40% of all PD patients practiced mindfulness, which is much higher than the 14.2% of adults in the United States that used (mindfulness) meditation in 2017^50^. Patients with milder disease were less likely to respond, and they perhaps experienced less stress and were also arguably less likely to resort to mindfulness. On the other hand, disease duration was not associated with the impact of stress, which is in keeping with common clinical observations of patients experiencing considerable levels of stress shortly after the diagnosis has been conveyed. Although mindfulness and meditation are increasingly visible in our society, it is also possible that the topic of our survey (stress and PD) motivated people who experience stress or use stress-reducing strategies to respond. Also, although the online nature of the survey made it possible to collect data of 5000 patients in a short time span, online studies are limited in several ways. It is possible that participants in remote areas or with limited internet access are underrepresented, and participants might be less engaged than in real-life study settings. Finally, although the reported effects of mindfulness are impressive (60.2% noticed improvement in depressed mood and 64.7% in anxiety), it is possible that these effects are (partly) driven by a placebo effect – which is particularly large in PD^51^.

In conclusion, our findings show that stress is an important topic for PD patients, that it has a considerable and detrimental influence on quality of life and on symptom severity, but that it is also potentially amendable to interventions aimed at reducing stress. Specifically, the significant beneficial effects that patients experienced from self-management strategies such as mindfulness and physical exercise encourages future trials into the clinical effects and underlying mechanisms of these therapies. Based on our findings, we expect that mindfulness interventions will have particularly large effects on depression and anxiety in PD.

## Methods

### Sample

We submitted our online survey to all eligible participants of Fox Insight, which is sponsored by The Michael J. Fox Foundation for Parkinson’s Research^52^. Participants affirmed an online statement of informed consent prior to the study, explaining the nature and length of the survey. Proceeding to fill out the survey was taken as consent to participate. Written consent was not obtained. Fox Insight is an online clinical study collecting participant-reported data about symptoms, daily activities, and other health factors from volunteers with and without PD, mostly in the United States. In addition to regular surveys, one-time surveys such as ours can be sent to the cohort.

### Demographics and clinical characteristics

We sent our survey with specific questions relating to stress and mindfulness to all 28,385 PD patients and 11,413 controls of the Fox Insight cohort. Socio-demographic data and clinical data of PD patients had been collected earlier, immediately after enrolment in the Fox Insight cohort, and included medication use and information about the diagnosis of PD. In addition, we used responses to several validated scales that had been completed earlier, including the Unified Parkinson’s Disease Rating Scale part II (MDS-UPDRS-II)^53^ to evaluate daily life symptom severity, the Non-Motor Symptoms Questionnaire (NMSQ)^54^, the Geriatric Depression Scale (GDS)^55^, Penn’s Parkinson’s Daily Activities Questionnaire-15 (PDAQ)^56^ assessing daily function dependent on cognition, and the Parkinson’s Disease Questionnaire-8 (PDQ)^57^ measuring perceived quality of life. After inclusion in the cohort, patients are asked to fill out these surveys at distinct time points throughout their individual Fox Insight participation. For respondents of our survey, we included responses to these routine surveys if they had been completed during the 12 months before receiving our additional questions. Given that the PDQ-8 was assessed more frequently, we used 6 months as a cut-off for this scale. Also, we only included scores for people who completed all items of these validated scales.

### Survey design

We developed two separate surveys: one for PD patients and another for controls. The patient survey consisted of an obligatory part, made up of six questions that took 5–10 min to complete, after which participants were allowed to choose whether they were willing to proceed and complete eight additional sets of questions (which took another 20–30 min). The control survey consisted of 10 sets of questions, which took 10–20 min to complete, and where PD-specific questions were omitted.

### Survey content

Table 1 describes the validated scales included in the optional part of both the patient and control survey. The Perceived Stress Scale (PSS)^58^ and Parkinson Anxiety Scale (PAS)^59^ were followed by the brooding subscale of the Ruminative Response Scale (RRS)^60^, measuring tendencies to repetitively think about the causes and consequences of one’s negative affect. We then proceeded with the Five-Facet Mindfulness Questionnaire (FFMQ)^61^ which assesses five traits that contribute to mindfulness: acting with awareness, describing, non-judging, non-reacting, and observing. Later, we added the Self-Compassion Scale (SCS)^62^, which measures the degree to which individuals display self-kindness against self-judgment, common humanity versus isolation, and mindfulness versus over-identification. Table 2 shows the remaining questions.

**Table 1:** Validated measures used in the one-time stress survey.

Scale	Items	Scoring options	Example of items
Perceived Stress Scale (PSS)	10 items	0 (never) to 4 (very often)	‘In the last month, how often have you felt that things were going your way?’
Parkinson Anxiety Scale (PAS)	12 items	0 (none or never) to 4 (severe or nearly always)	‘To what extent did you experience episodes of panic or intense fear in the past four weeks?’
Ruminative Response Scale (brooding subscale; RRS)	5 items	0 (almost never) to 3 (almost always)	‘How often do you think: what am I doing to deserve this’
Five-Facet Mindfulness Questionnaire (FFMQ)	15 items	1 (never or very rarely true) to 5 (very often or always true)	‘I notice how foods and drinks affect my thoughts, bodily sensations, and emotions’
Self-Compassion Scale (SCS)	12 items	1 (almost never) to 5 (almost always)	‘I’m disapproving and judgmental about my own flaws and inadequacies’

This table gives an overview of the validated scales we used in our survey. We show the number of items per scale, the response options given (all on a Likert-scale), and for each scale an example of the items.

**Table 2:** Additional questions in the one-time stress survey.

Question	Items	Scoring options
**How does stress or anxiety affect the following Parkinson symptoms in your experience?**	• Tremor (trembling or shaking) • Problems with walking (including freezing)	1 (symptom worsens a lot) to 9 (symptom improves a lot); 5 = no effect.
**If you do not experience this symptom, please choose “not applicable”**.	• Slowness of movements (for example when writing or getting dressed) • Excessive movements (dyskinesia, not tremor) • Depressed mood • Sleeping difficulties	
**a) For each of the following possibilities, please state how often you have used them over the last three months**. ^1^	• Physical therapy • Physical exercise (for example walking, cycling, swimming, sports)	a) 1 (never) to 7 (several times a day)
**b) For each option, please state how effective it is to reduce stress** ^1^	• Relaxation exercise (for example yoga, Pilates, Tai Chi) • Mindfulness • Other types of meditation	b) 0 (not at all) to 10 (excellent)
**Some people report that certain activities reduce or increase their motor symptoms. Motor symptoms include tremor, muscle stiffness, gait problems, or movement slowness. For each of the following activities, please state the change you typically observe in your Parkinson motor symptoms**.	• Social stress (example: talking in a group, or when being evaluated by others) • Conflicts in relationships (example: a row with your partner or boss) • Concentration (example: reading a book, or playing an instrument)	
**Choose “not applicable” if you do not experience these activities**.	• Time pressure (example: being late for an appointment) • Worrying (example: thinking about financial troubles) • Excitement (example: after receiving good news) • Doing something you really enjoy (example: painting, gardening, or another hobby)	1 (symptom worsens a lot) to 9 (symptom improves a lot); 5 = no effect.
**We would like to evaluate your experience with mindfulness. What are your reasons for not doing mindfulness at the moment? You can choose more than one option. If nothing applies, you do not have to fill out anything**.^1^	• I have never thought about this option, but would be interested in doing mindfulness • I don’t experience any stress • I don’t believe mindfulness will work for me • I don’t have the time or the energy to participate in a course • There are no courses near my home • I find it too expensive • I don’t like group sessions, but would be interested in individual or online courses	Multiple choice
**It is possible that you have noticed changes in your Parkinson symptoms since you started mindfulness. How much has mindfulness changed each of the following symptoms?**	• Tremor (trembling or shaking) • Problems with walking (including freezing) • Slowness of movements (for example when writing or getting dressed)	1 (symptom worsens a lot) to 9 (symptom improves a lot); 5 = no effect.
**If you do not experience a symptom, choose “not applicable”**.	• Excessive movements (dyskinesia, not tremor) • Depressed mood • Sleeping difficulties	

^1^**:** questions were included in the control survey as well.

This table gives an overview of the questions we used in our patient survey. The first column shows the exact question, the second column shows items within that question, and the last column shows response options, differing per question.

### Statistical analysis

Data used in the preparation of this article were obtained from the Fox Insight database (https://foxinsight-info.michaeljfox.org/insight/explore/insight.jsp) on 01/02/2020. Data was then loaded into IBM SPSS Statistics 23 to calculate sum-scores of validated scales and to perform statistical testing. The survey was designed to answer several (related) questions regarding the interplay between PD, stress, and mindfulness. We corrected for the number of comparisons (e.g., symptoms or questionnaires) using a Bonferroni correction, and report effects with 95% confidence intervals. First, separately for PD patients and controls, we compared all variables between responders and (invited) non-responders, using independent-sample *t*-tests for continuous variables and *χ*^2^-tests for categorical variables. Here we did not correct for multiple comparisons, since statistical tests were meant to describe the cohort. Non-responders were defined as people who had not filled out any of the survey questions (Table 3). Second, for the responders, we calculated group differences (patients versus controls) using univariate ANCOVA’s with age and sex as covariates, given that PD responders were significantly older and consisted of a larger proportion of men than control responders (Table 3). We corrected for multiple comparisons using an adjusted statistical threshold of *p* < 0.05/9 = 0.0056. Third, in the PD group, we explored how stress level (PSS score) related to the other four questionnaires (Table 1) and to two indexes of disease severity (MDS-UPDRS-II and disease duration), using Pearson correlation coefficients with an adjusted statistical threshold of *p* < 0.05/6 = 0.0083. Fourth, in the PD group, we tested for the (self-reported) effect of stress on six motor and non-motor symptoms using one-sample *t*-tests (where a test value of 5 indicates no change) with an adjusted statistical threshold of *p* < 0.05/6 = 0.0083. Fifth, given our focus on mindfulness, we assessed whether PD mindfulness users differed from non-users in stress level (PSS score), anxiety (PAS score), rumination (RRS score), self-compassion (SCS score) and dispositional mindfulness (FFMQ score), disease duration and use of dopaminergic medication, using independent-sample *t*-tests with ‘mindfulness practice’ (yes/no) as grouping variable, and an adjusted statistical threshold of *p* < 0.05/7 = 0.0071. Finally, we studied the self-reported effect of mindfulness on six PD symptoms, using one-sample *t*-tests with test value 5 (indicating no change) and an adjusted statistical threshold of *p* < 0.05/6 = 0.0083. We divided the group of mindfulness users according to the frequency with which they use mindfulness, and calculated Spearman’s correlations between frequency of mindfulness use perceived change in symptom severity, again using a statistical threshold of *p* < 0.05/6 = 0.0083.

**Table 3:** Demographics of the study population.

	Parkinson’s patients				Controls				Difference
	Responders (*n* = 5000)	Number of valid responses	Non-responders (*n* = 23381)	Responders vs. non-responders	Responders (*n* = 1292)	Number of valid responses	Non-responders (*n* = 11413)	Responders vs. non-responders	PD vs. controls
Age (years)	67.3 (8.9)	5000	66.4 (10.1)	*p* = 0.819	60.8 (13.0)	1292	57.4 (14.0)	*p* = 0.000*	*p* = 0.000*
Sex (% female)	48.6%	4982	44.7%	*p* = 0.971	78.0%	1288	77.0%	*p* = 0.386	*p* = 0.000*
Stress level in daily life (10-point scale)	4.4 (1.9)	5000			4.6 (1.9)	1292			*p* = 0.000*
Perceived Stress Scale	15.9 (7.5)	3127			15.3 (7.0)	1292			*p* = 0.000*^,^^1^
Parkinson Anxiety Scale	13.6 (8.9)	2899			11.7 (8.4)	1292			*p* = 0.000*
Five Facet Mindfulness Questionnaire	51.5 (8.0)	2899			52.7 (8.4)	1292			*p* = 0.000*
Self-Compassion Scale	41.0 (9.2)	2899			40.5 (9.6)	1292			*p* = 0.150 ^1^
Ruminative Response Scale	3.5 (3.0)	2899			3.7 (2.9)	1292			*p* = 0.219 ^1^
Parkinson Disease Questionnaire	14.7 (13.7)	4784	19.7 (16.9)	*p* = 0.389					
Unified Parkinson’s Disease Rating Scale II	22.4 (7.2)	4742	24.5 (8.3)	*p* = 0.006*					
Parkinson’s Daily Activities Questionnaire	50.5 (9.5)	4710	49.4 (10.7)	*p* = 0.000*					
Non-Motor Symptoms Questionnaire	10.9 (5.3)	4838	11.2 (5.4)	*p* = 0.126					
Geriatric Depression Scale	4.1 (3.7)	4369	4.6 (4.0)	*p* = 0.010*	2.6 (3.0)	1237	3.4 (3.5)	*p* = 0.000*	*p* = 0.000*
Disease duration (years)	5.9 (5.2)	4966	6.3 (5.9)	*p* = 0.631					
Use of levodopa or dopamine agonists (%)	92.8%	4935	93.2%	*p* = 0.738					

^1^: significant group*sex interaction, reported *p*-values are related to main effect of group, controlling for sex.

This table shows mean (SD) or percentages for demographic and clinical characteristics. Column 3 and 7 show the number of valid responses for PD and control respondents, since some of the baseline data was not available, and there was an optional part in our patient survey. Columns 5 and 9 show *p*-values of differences between invited non-responders and all responders, for continuous variables measured with *t*-tests and for categorical variables with *χ*^2^-tests. We indicated all significant differences of *p* < 0.05 with *.

### Ethical approval

The Fox Insight informed consent procedure and study protocol are approved by the New England Independent Review Board (IRB#: 120160179, Legacy IRB#: 14–236).

### Reporting summary

Further information on research design is available in the Nature Research Reporting Summary linked to this article.

## Supplementary information

Reporting Summary

## Data Availability

All individual data collected through the Fox Insight online clinical study is available on the Fox Insight database (https://foxinsight-info.michaeljfox.org/insight/explore/insight.jsp). Qualified researchers, interested in Parkinson’s disease or related research, can access Fox Insight data upon account registration, completion of a Data Use Agreement (DUA), acknowledgement of the study publication policy, and review by the Data and Publications Committee.
